# Do health professionals have a prototype concept of disease? The answer is *no*

**DOI:** 10.1186/s13010-017-0047-7

**Published:** 2017-09-11

**Authors:** Bjørn Hofmann

**Affiliations:** 1 Institute for the Health Sciences, The Norwegian University Science and Technology, PO Box 1, N-2802 Gjøvik, Norway; 2 Centre for Medical Ethics at the University of Oslo, Oslo, Norway

**Keywords:** Disease, Concept, Prototype, Falsification, Language

## Abstract

**Background:**

Health and disease are core concepts in health care and have attracted substantial interest and controversy. In recent and interesting contributions to the debate it has been argued that the challenges with the concept of disease can be resolved by a prototype concept of disease. As a robin is a more prototypical of a bird than a penguin, some diseases are more prototypical than others. If disease is a prototype concept, it would change nosology, but also health care and the study of health and disease. However, the statement that “disease is a prototype concept” forms an empirically testable hypothesis. Therefore, this study aims to test the hypothesis that health professionals have a prototype concept of disease.

**Methods:**

Two hundred twenty-three health care professionals in Norway were invited to participate in a survey where they were asked to rank a wide range of diseases according to how typical they considered them to be as diseases. Results were analysed with descriptive statistics.

**Results:**

The response rate was 90%. Lung cancer, leukemia, colon cancer, myocardial infarction, and AIDS are the diseases ranged to be most typical, while homosexuality, pregnancy, drapetomania, dissidence, and nostalgia are considered to be the least typical diseases. The results also show that the answers to how typical various diseases are vary greatly, even amongst a relatively homogenous group of health professionals.

**Conclusion:**

This study falsifies the hypothesis that disease is a prototype concept for health professionals. This has implications for the debate on core concepts for health care. If health professionals do not have a prototype concept of disease, it is unlikely that there is a prototype concept of disease in general. Consequently, nosologies should not be based on prototypes.

## Background

Health and disease are core concepts in health care and have attracted substantial interest and controversy [1–16]. The eager to define disease is fuelled by the strong needs for demarcation, i.e., to differentiate those who are entitled to attention, treatment, and care by health professionals, who are freed from duties, such as work (sick leave), and who are entitled to economic support from those who are not [17–19]. As such, the concept of disease is of great theoretical and practical import for health care. However, the concept of disease poses profound (ontological, epistemological, and ethical) challenges, yet not settled [20], and defining disease has turned out to be demanding [16]. Some even argue that it is impossible or even quite unnecessary [8, 21].

It has been argued that these difficulties are due to confusion between the category of disease (i.e., disease in general), the disease entity (i.e., the disease type, e.g., infectious disease), and the specific instance of disease (i.e., the disease token, e.g., Mr. Hanson having an infection) [22]. The first is a topic for philosophy (metanosology), the second for nosology, and the third for diagnostics.

According to the “classical concept” view the challenges with definitions result from trying to define the term “disease” by searching for a set of essential features common to all diseases [23]. When recognizing disease, the main challenges consists in identifying a series of defining features qualifying for falling under a certain concept [22]. However, this does rarely work. No such common features have been identified. Accordingly, it is suggested that we have to apply a “non-classical concept” view. I. e., we have to turn to a concept of disease which is not based on a set of common features of its members (i.e., individual diseases), it is argued, but on a few best examples of the category, which are called its prototypes [22, 24–30]. A condition is a disease if it resembles its prototype. Accordingly, “the prototype resemblance theory of disease … promises to lead the philosophy of disease out of its impasse and to stimulate innovative inquiry” [22]. In particular, a prototype theory of disease has been promoted recently [11, 22]. If disease is a prototype concept, it would change nosology, but also health care and the study of health and disease. We should stop wasting our time on theoretical deliberations and use our energy for empirical studies.

Therefore it is important to test the statement that “disease is a prototype concept.” And there exist empirical studies on the prototype theory of concepts exists with regard to specific disease entities and symptoms [31–33]. However, the claim that disease is a prototype concept (in general) has not been tested. One reason for this may be the “two cultures” divide in the studies of health and disease: theoretical and empirical. However, as theoretical approaches make empirically testable claims, the divide should be bridged.

Therefore, if the statement that “disease is a prototype concept” is true and people have a prototype conception of disease they should range specific diseases fairly similarly with respect to how typical they believe the diseases are. Accordingly, the null hypothesis of this study is that disease is a prototype concept. If people similarly agree on how various diseases are typical, in the same way as they agree that sparrows, and not ostriches, are typical birds, it supports the null hypothesis. If not, the null hypothesis is falsified.

## Methods

A survey was developed comprising a mixture of well-established uncontroversial diseases and controversial conditions. For comparative reasons the selection is based on a study of health professionals conceptions of prestige related to diseases [34].

The respondents were asked to rate whether they found the diseases more or less typical in the same manner as they would find different birds, such as sparrows versus ostriches, more or less typical of birds. Answers were given from 0 to 10, where 0 was untypical, and 10 was very typical.

A preparatory pilot study was performed. The survey was used for a group (of 21) persons of the general population and for a group of health professionals (23). The pilot prompted clarification of some questions in the revised survey. As the test on the general population gave very disperse results, it was decided to use the survey for health professionals. The final survey, which was in Norwegian, was printed on white paper (1 page, 2-sided).

The survey was distributed to 223 health related professionals while they were attending university courses or seminars at the University of Oslo, Faculty of Medicine, during the spring terms of 2010–2014.

The respondents were categorized as MD, RN, physiotherapists, psychologist, dentist, biologist, and other, where the latter category contained epidemiologists, social scientists, philosophers, and professionals with background from other humanities. The respondents either had a Ba (87) or a Ma (113) level education.

The data were registered manually in Microsoft Excel 2010. Statistical analysis was performed with SPSS. Descriptive analyses were used to describe the materials. Differences in answers with respect to education level was tested with t-test and differences between professions and between years of response was tested with ANOVA. For all tests *p* < 0.01 in order to reduce the risk of type-1 error as the number of tests were quite high.

Responses were anonymous and participation was voluntary. No personal data traceable to individual participants were registered, and the study was thus not subject to REC/IRB or Data Protection approval, in accordance with Norwegian law. Participants consented to participating in the study by filling in and handing in the form.

## Results

One hundred thirteen out of 129 respondents with Ma level education participated and 87 out of 94 with Ba level education participated. Most of the professionals with Ma were MDs (43.3%), psychologists (14.4%), and RNs (11.3), and most of the professionals with Ba were RNs (42.3%) and physiotherapists (22.7%). The response rate for persons with Ma level education was 87.6% and 92.6% for Ba level educations, while the overall response rate was 90.0%.

The overall ratings for the various diseases are shown in Table 1. Lung cancer, leukemia, colon cancer, MI, and AIDS are the diseases ranged to be most typical, while homosexuality, pregnancy, drapetomania, dissidence, and nostalgia are considered to be the least typical diseases. Aspergers syndrome, ectopic pregnancy, femur fracture, obesity, candida are the conditions with the greatest divergence among the respondents.

**Table 1:** Average ratings for all respondents ranged according to mean score

Disease/Condition	Mean	SD
Lung cancer	9.91	0.49
Leukemia	9.85	0.78
Colon cancer	9.82	0.80
Myocardial Infarction	9.79	0.78
AIDS	9.74	0.81
Renal failure	9.72	1.06
Multiple Sclerosis	9.69	0.90
Diabetes	9.36	1.53
Appendicitis	9.35	1.76
Reumatoid arthritis	9.33	1.32
Schizophrenia	9.20	1.42
Bechterews disease	9.16	1.54
Angina Pectoris	9.02	1.62
Rupture of the spleen	8.98	2.42
Asthma	8.97	1.70
Liver cirrhosis	8.87	2.08
Dementia	8.79	1.97
Ulcus duodeni	8.69	2.10
Psoriasis	8.64	2.09
Cataract	8.60	2.26
Twar (pneumonia clamydiae)	8.59	2.14
Kidney stone	8.49	2.59
Anorexia	7.76	2.29
Femur fracture	7.73	3.66
Prolapse	7.64	2.71
Borderline personality disorder	7.60^a, b^	2.28
Anxiety neurosis	7.46^a^	2.33
Aspergers syndrome	6.97	5.94
Eating disorder	6.63	3.12
Ectopic pregnancy	6.23	3.80
Lumbago	6.12	3.06
Alcoholism	6.11	2.73
ADHD	5.95^a^	2.74
ME/CFS	5.91^a^	2.55
Self affliction	5.90	2.97
Opioid dependency	5.68	3.17
Erectile dysfunction	5.67	2.67
Fibromyalgia	5.64^a^	2.47
Candida	5.56	3.38
Gulf war syndrome	5.44	2.83
Irritable Bowel Syndrome	5.33	2.88
Agorafobia	4.67^a^	2.87
Neurasthenia	4.59	2.9
Obesity	4.54	3.58
Dysmorphophobia	3.73	2.96
Gambling dependence	3.62	2.76
Alergia to electric waves	3.49^a, b^	2.84
Infertility	3.36	3.19
Premenstrual syndrome (PMS)	2.85	2.86
Cleptomaina	2.82	2.64
Spontaneous abortion	2.79	3.23
Internet pornography dependence	2.78	2.84
Shopping dependency	1.99	2.42
Coffein dependency	1.58	2.21
Coca-Cola dependency	1.40	2.12
Menopause	1.26	2.41
Media-victim-syndrome	1.23	1.70
Nostalgia	0.74	1.41
Dissidence	0.74	1.45
Drapetomania	0.73	1.66
Pregnancy	0.40	1.35
Homosexuality	0.26	0.80

^a^Statistical significant difference between respondents with Ba and Ma level education (*t*-test, *p* < .01)

^b^Statistical significant difference between the years of response (sinking scoring with time) (ANOVA, *p* < .01)

For several conditions there are substantial variations in ratings. Table 2 shows the standard deviation for the various conditions for the respondents with Ba and Ma level education.

**Table 2:** Standard deviations for participants with Ba and Ma level education

	Ma level education	Ba level education
Aspergers syndrome	8.95	2.92
Femur fracture	3.65	3.66
Ectopic pregnancy	3.57	4.03
Candida	3.33	3.43
Spontaneous abortion	3.25	3.20
Infertility	3.13	3.25
Opioid dependency	3.13	3.20
Dysmorphophobia	3.04	2.88
Eating disorder	2.95	3.28
Agorafobia	2.95	2.78
Self affliction	2.94	2.99
Obesity	2.93	4.23
Lumbago	2.85	3.27
Internet pornography dependency	2.82	2.85
Premenstrual syndrome (PMS)	2.80	2.91
Alcoholism	2.75	2.70
Erectile dysfunction	2.72	2.62
Irritable Bowel Syndrome	2.72	3.04
Neurasthenia	2.69	3.11
Gambling	2.68	2.84
Alergia to electric waves	2.66	3.02
ADHD	2.60	2.87
Cleptomaina	2.59	2.69
Gulf war syndrome	2.59	3.07
Anorexia	2.54	2.04
ME/CFS	2.53	2.56
Coffein dependency	2.52	1.89
Prolapse	2.47	2.94
Anxiety neurosis	2.45	2.21
Fibromyalgia	2.40	2.53
Kidney stone	2.37	2.81
Menopause	2.36	2.45
Borderline personality disorder	2.32	2.23
Coca-Cola dependency	2.24	2.00
Shopping dependency	2.23	2.60
Twar (pneumonia clamydiae)	2.18	2.10
Rupture of the spleen	2.18	2.65
Cataract	2.00	2.51
Dementia	1.98	1.95
Ulcus duodeni	1.90	2.29
Psoriasis	1.87	2.30
Angina Pectoris	1.83	1.41
Bechterews disease	1.81	1.26
Liver cirrhosis	1.74	2.41
Media-victim-syndrome	1.66	1.74
Asthma	1.60	1.80
Nostalgia	1.57	1.24
Schizophrenia	1.50	1.34
Reumatoid arthritis	1.43	1.20
Pregnancy	1.37	1.32
Appendicitis	1.37	2.14
Renal failure	1.25	0.87
Diabetes	1.24	1.81
Myocardial Infarction	1.19	0.37
Dissidence	1.19	1.71
Colon cancer	1.15	0.45
Homosexuality	1.11	0.48
Multiple Sclerosis	1.02	0.78
Drapetomania	1.02	2.30
AIDS	0.75	0.87
Leukemia	0.57	0.99
Lung cancer	0.44	0.54

Table 3 illustrates the values and standard deviations for different professions.

**Table 3:** Mean values and standard deviations for various professions for those conditions where the difference (in mean values) for the professions was highest

	MD		Psychologists		RN	
	Mean	SD	Mean	SD	Mean	SD
Agorafobia (*p* < 0.001)	5.78	2.87	8.53	1.89	3.69	1.41
Cleptomaina (*p* = 0.007)	3.02	2.69	5.61	2.81	1.18	0.92
Ectopic pregnancy (*p* = 0.004)	7.95	2.93	6.63	4.53	3.78	1.89
Internet pornography dependency	2.61	2.61	5.17	3.56	1	0.66
Alcoholism	6.5	2.51	8.25	2.38	4.1	1.92
Gambling dependency (*p* = 0.001)	3.65	2.46	6.47	2.56	2.48	0.91
Shopping dependency	1.79	2.3	4.37	2.81	0.42	0.65
Coffein dependency (*p* = 0.001)	2.34	2.61	3.95	3.72	0.2	0.59
Angina Pectoris (*p* = 0.002)	8.99	1.62	6.21	3.11	9.65	0.68
Femur fracture	8.92	2.65	6.8	4.35	5.68	1.84
Aspergers syndrome	9.16	14.03	6.9	2.78	6.43	1.45
Spontaneous abortion	3.2	3.45	0.7	1.75	3.06	1.99
ME/CFS (*p* = 0.004)	4.65	2.44	7.05	2.2	6.97	1.13
Alergia to electric waves	2.08	2.44	4.38	2.81	2.05	1.81
Dysmorphophobia (*p* = 0.003)	4.16	2.87	6.54	2.43	4.37	0.98
Fibromyalgia	4.91	2.38	6.6	2.51	4.13	1
Neurasthenia	3.68	2.41	5.83	2.04	5.75	1.12
Obesity	4.66	2.8	6.8	3.24	4.77	1.65
Twar (pneumonia clamydiae)	8.83	2.39	6.58	3.14	8.1	0.98
Opioid dependency	6.15	2.96	6.81	2.57	4.77	1.86
Irritable Bowel Syndrome	4.96	2.12	7.03	2.71	5.98	2.02
Nostalgia (*p* = 0.007)	0.45	0.97	1.32	1.79	0.3	0.2

Where there was a statistical significant difference the *p*-values are provided in parentheses (ANOVA)

## Discussion

Although there is some agreement, there is considerable divergence with respect to how typical various diseases are considered to be. This corresponds well with a study asking people whether they consider various states to be disease or not and whether they think the states should be treated with public tax revenue. Due to the divergence in responses, none of the diseases in the survey qualify as prototypes. Moreover, if there exists a disease prototype not included in this study, one would expect that comparing the diseases included in the study to this prototype would result in rather homogeneous assessments, especially if the prototype theory is expected to have valuable practical implications as claimed by its proponents.

As with all surveys, there are many sources of errors. E.g., the respondents were all professionals under education, either for a Ma or PhD, and all were attending university courses. Hence, they may be a selected group and the sample may give substantial bias resulting from convenience sampling. However, as indicated by the pilot study, it seems that an unselected group of professionals or non-professionals would have given even more divergent results. The coherence with other findings also indicate that the risk of bias due to convenience sampling may be limited. The intention of this study was to see if a fairly homogenous group of health professionals have a prototype conception of disease.

Moreover, a series of changes may have occurred during the data collection period (2010–2014). However, the results do not reveal any systematic changes over this time period, although some changes are statistically significant. If a disease prototype theory had any practical value, one would expect it to be fairly stable over a time period of some years, especially as the prestige of diseases turn out to be surprisingly stable [34].

It may of course also be that Norwegian health professionals are special(ly confused), or that the wrong cases were used. However, if a prototype concept of disease is not relevant to the selected cases in this study, one could argue that it is of little relevance to health care, to the philosophy of medicine, and to health policy.

Respondents may of course also misinterpret disease entities. IBS and fibromyalgia may be conceived of differently by different respondents, who may also be ignorant about drapetomania. However, explanations to rare conditions were given, and it is difficult to explain all the variation from misinterpretations. Correspondingly, some respondents may have misunderstood the phrasing “how typical the disease entities are as a disease,” e.g. as “how much do you think the disease entities deserve to be diseases” etc. It may also be that participants in courses with the topic “what is disease” are particularly uncertain about the disease concept. However, the pilot studies showed that the incoherent conception of disease typicality was even more outspoken in persons from the general population and from other health professions.

Likewise, it may be argued that none of the given disease entities (types) are close to the prototype of disease (in general). This appears to be a highly reasonable objection. Moreover, it may be argued there exist no prototypes for the various disease entities of the survey, but only for disease in general. However, in both cases the respondents are presupposed to share a common prototype concept of disease, and we would expect that the respondents’ variation is rather small. If you cannot observe the prototype in practice, it is of limited value.

In the same vein one could argue that the variations are not that big and that there is significant agreement in the results. Prototype theory of concepts give some variation in the concept space [35]. This is also a relevant objection. However, the variations are so substantial that they may not do the job of moving the debate on the concept of disease out of its alleged impasse. There are of course many ways to measure prototypicality [36], and it can be argued that the method here is too crude. Again, this is a reasonable objection, but if the prototype conception cannot be applied in a straight forward way, it may not lead us forward, as promised.

Correspondingly, one may argue that there is no prototype of a general concept of disease, but several prototypes to specific sub-groups of disease, e.g., of infections, injuries, or organ failures [30]. This reduces the potential to and implications of leading “the philosophy of medicine out of its impasse.” Moreover, it poses the question of how to delimit the areas where the prototype conceptions of disease reside. Most importantly, even with delimited prototype concepts of disease, one would expect coherent conceptions within these fields. Even with “different definitions for different contexts” [30] the conceptions should be similar within a given context.

Another objection is that there is a significant variation in the application of the prototype (in this study), but not in the prototype itself. This may of course very well be so, but the problem with this argument is that it undermines the relevance of disease as a prototype concept. If prototype concepts do not give regular uses or if you cannot assess whether they exist in interested and reflected health professionals, it may be argued that it becomes of little interest whether disease is a prototype concept in the first place.

A more general argument against this study is that the nature of the disease concept cannot, in principle, be resolved by empirical studies, but only by philosophical analysis (of scientific theory of pathology). As impossible as it is to refute in chemistry the possible theory that "chemical element" is a prototype concept by empirically asking "chemistry professionals" for ranking substances according to typicality, as impossible it is in medicine to test and refute the general prototype theory of the concept of disease empirically. Hence, one could dismiss the hypothesis on theoretical grounds. Nonetheless, it is appropriate to test empirical claims with empirical methods, and in this case it supports continued philosophical endeavour.

A side effect of the study is that it also falsifies the hypothesis that the surprisingly steady hierarchy found among medical doctors in their prestige ranking [34] stems from how the typicality of disease entities (and not as much from their prestige). Hence, prestige ranking of diseases, cannot be explained by the protopyicality of the diseases.

No doubt, the prototype theory of the concept of disease is plausible. Concepts have been considered to be prototypes (or stereotypes) in the philosophy of language. Wittgenstein and a series of psychologists [24, 37, 38] have argued that what is distinguished among the members of a category X is not an invariant property found in each and every instantiation of X, but rather many properties that tend to co-occur in their presentations to our consciousness. When we are exposed to Xs (by sensations or thoughts) we learn how to identify Xs according to the display frequency of their co-incident properties. This phenomenon of prototype recognition has been described by Andy Clark (1998) as: “the notion of the statistical central tendency of a body of concrete exemplars. Such central tendency is calculated by treating each concrete example as a set of co-occurring features and generating (as the prototype) a kind of artificial exemplar that combines the statistically most salient features. …Concrete exemplars and rich worldly experience are still crucial, but they act as sources of data from which these artificial prototypes are constructed. Novel cases are then judged to fall under a concept…according to the distance separating the set of features they exhibit from the prototypical feature complex.” [39]. However, prototype theory of concepts has also been criticized and moderated [23]. Entering this debate is beyond the scope of this article.

Here I only have empirically tested (and falsified) the null hypothesis. If the results reveal the true conceptions of the respondents, there exists no prototype concept of disease among health professionals in general. There may of course subgroups that have clear prototype concepts of disease, however those are not MDs, psychologists, and RNs in general. Again, the concept of disease may well be a prototype concept outside these groups, but if these groups are excluded, the value of a prototype theory of disease is limited. Hence, despite the limited extension of this survey and its flaws, it falsifies the general hypothesis that health professionals have a prototype concept of disease.

Prototype theory may of course still be of some theoretical value as specific disease entities may very well be prototype concepts. Other concept oriented approaches, such as concept mapping [40, 41], may also be fruitful. However, it is far from clear that the prototype theory of disease will (re)solve the many conceptual and normative challenges as promised by its proponents [22].

## Conclusion

This study has demonstrated that there exists no clear prototype concept of disease among health professionals in general and that this approach does not keep its promise “to lead the philosophy of disease out of its impasse.” Consequently, it is not likely that prototype concepts of disease will solve conceptual or nosologies challenges. Moreover, the study encourages empirical testing even in theoretical work on health and disease. Bridges between theoretical and empirical work can enrich both “cultures.”

## Abbreviations

ADHD: Attention Deficit Hyperactivity Disorder
IBS: Irritable Bowel Syndrome
ME/CFS: Myalgic encephalomyelitis (ME)/Chronic Fatigue Syndrome
MI: Myocardial Infarction
